# Long-Term Results of Modified Catheterization Technique in the Treatment of CE Type 2 and 3b Liver Hydatid Cysts

**DOI:** 10.1007/s00270-025-03976-1

**Published:** 2025-02-14

**Authors:** Okan Akhan, Yakup Özbay, Emre Ünal, Ergun Karaagaoglu, Turkmen Turan Çiftçi, Devrim Akıncı

**Affiliations:** 1 Department of Radiology, Faculty of Medicine, Hacettepe University, Ankara, Turkey; 2 Department of Biostatistic, Faculty of Medicine, Hacettepe University, Ankara, Turkey

**Keywords:** Mo-CAT, Aggressive irrigation, Liver hydatid cyst, CE2 and CE3b, Surgery

## Abstract

**Purpose:**

To evaluate the long-term results of modified catheterization technique (Mo-CAT) for percutaneous treatment of liver CE2/CE3b hydatid disease in a large series.

**Materials and Methods:**

A total of 119 patients (F/M:73/59) and 132 liver CE2 and CE3b cysts who underwent percutaneous treatment by Mo-CAT from 2009 to 2020 were included in the study. Patients’ age ranges from 8 to 78 years (mean: 39 years). Volume changes of all cysts after the procedure, success and complication rates, duration of hospital stay, catheterization time and recurrence rates were recorded. Technical success was defined as successful catheter introduction into the CE. Clinical success was defined as cases with no mortality and no recurrence.

**Results:**

Among all patients, the mean reduction in the cyst volume was 65.84% (range 6.29–100%). The mean length of hospital stay was 3.88 ± 4.73 days (range 1–36 days). A total of 107 (89.9%) of 119 patients were discharged from the hospital in first the week after the procedure. Major complications were observed in 12 of 119 patients (10.08%) and 12 out of 132 cysts (9.09%). Recurrence was detected in 6 (4.5%) cysts in 6 patients (4.5%) who needed additional procedures. Among all 119 patients, the mean follow-up duration was 51.66 ± 35.56 months (median, 49.00 months; range 0–131 months).

**Conclusions:**

Treatment of liver CE2/3b with Mo-CAT appears to be a safe, reliable and efficient technique which is associated with low recurrence and complication rates.

**Graphical Abstract:**

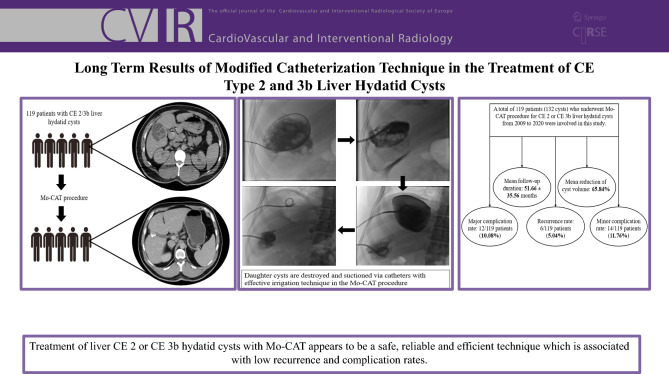

## Introduction

Cystic echinococcosis (CE), traditionally known as hydatid cyst, is a common public health problem in several parts of the globe [1–3]. CE can be classified according to the criteria of Gharbi or the World Health Organization (WHO). According to the WHO classification, CE1 and CE2 refer to active stage of the parasite, CE3 refers to transitional stage and CE4 and CE5 refer to inactive/degenerative stage [4]. While first three groups need to be treated, CE4 and C5 groups do not need to be treated.

There are four management options in the treatment of uncomplicated liver cystic echinococcosis. These include medical treatment, surgery, percutaneous treatment and “wait and watch” approach [4]. Wait and watch approach is dedicated for the follow-up of CE 4 and CE 5 cysts which are accepted to be inactive hydatid cyst. Surgery which used to be the traditional choice of treatment for liver CE2/CE3b cysts is associated with some serious outcome such as high morbidity, mortality and recurrence rates with long duration of hospital stay [5–8].

There are mainly three accepted percutaneous techniques for the treatment of alive liver CE as puncture, aspiration, injection, and re-aspiration (PAIR), standard catheterization (S-CAT) and modified catheterization (Mo-CAT) techniques. PAIR has first been described in 1986 [9, 10] which has become a treatment alternative to surgery as it is associated with lower morbidity, mortality and recurrence rates and shorter hospital stay for the treatment of CE1/CE3a cysts [5, 11]. S-CAT was first described in 1993 and has been widely used since then [11–15]. PAIR should be preferred to S-CAT for treatment of liver CE1/CE3a cysts due to lower rates of major complications and shorter length of hospital stay. However, S-CAT should be employed when cysto-biliary fistulas or any technical difficulty is detected during PAIR [16].

Recurrence rates are found to be high up to 61% when CE2/CE3b cysts are treated by PAIR or S-CAT [17–19]. Therefore, searching for a new percutaneous technique started since the early 90s to treat CE2/CE3b cysts ended up with the description of Mo-CAT [19–21].

The aim of the current study is to investigate the efficacy and safety of Mo-CAT technique for the treatment of liver CE2 and CE3b cysts in a large series and evaluate the long-term results.

## Methods

### Population and Definition

This is a retrospective observational study which was approved by the ethics board. Informed consents from all patients were obtained before the procedures. A total of 119 patients with 132 liver cysts were treated by Mo-CAT technique from February 2009 to September 2020 followed up until February 2021.

Our inclusion criteria were patients with ultrasonographic findings consistent with CE2 (multiseptated cyst resembling a rosette) or CE3b (Cyst with daughter cysts in solid matrix) liver hydatid cysts according to WHO classification who were willingness to undergo to the procedure (or if the patient is a child, whose parent’s permission is acceptable) and age more than 8 years. Exclusion criteria included liver CE1/CE3a with CE4/CE5 besides all extrahepatic CE as well as patients younger than 8 years, women who were pregnant at the time of diagnosis, patients with all clinical diseases involving a life-threating risk such as sepsis or severe organ failure.

Technical success was defined as successful catheter placement into the cyst and completion of all steps for Mo-CAT procedure. Clinical success was defined as cases with no mortality and no recurrence. Complications were divided as minor and major and graded based on the CIRSE classification of complications [22]. The detection of daughter cysts in the mother cyst during follow-up was considered as an indicator of recurrence. Patient characteristics (age, gender), cyst characteristics (single or multiple cysts, volume, CE type), duration of follow-up and length of hospital stay, complications and recurrence rates were recorded.

### Procedure Preparation

Abdominal US and chest X-ray of all the patients were obtained before the procedures. Liver MRI/MRCP was also performed, if needed, to confirm the diagnosis of liver CE2/CE3b.

All patients were continuously given adjunctive Albendazole at 10 mg/kg/day for 1–7 days before and 4 weeks following the procedure in order to reduce the probability of developing seeding cysts due to spillage. The patients’ complete blood count, coagulation parameters and liver function tests were reviewed before the procedure. All procedures were performed under US and fluoroscopic guidance by experienced interventional radiologists in a university hospital under general anaesthesia due to the possible risk of lethal anaphylaxis.

### Technique

**Fig. 1:**
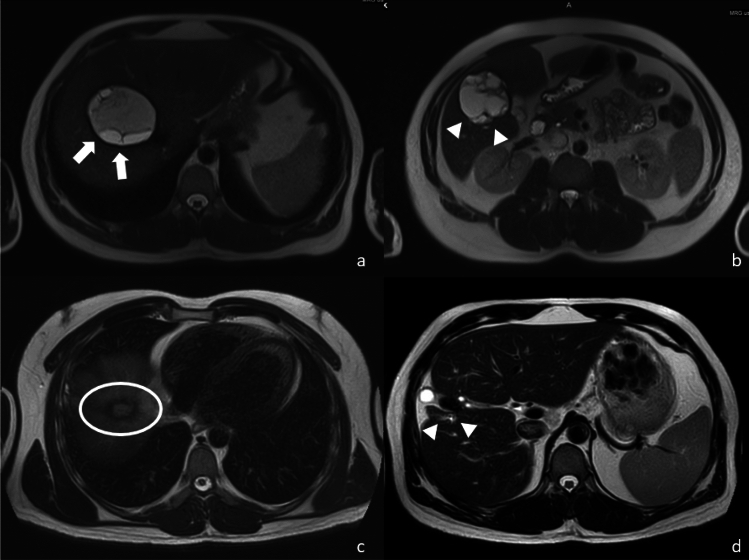
**a** A 34-year-old male patient who applied to the hospital presented with right upper quadrant pain, abdominal MRI revealed CE type 3b hydatid cysts located in segment 8 (a, white arrows) and segment 5 (b, white arrowheads) of liver. 18 months later, control MRI demonstrated that there was no active cyst or recurrence at previous cysts sites but some sequelae changes and calcifications (c, white circles; d; white arrowheads). **b** During the Mo-CAT procedure of the same patient, after inserting of Amplatz wire (a, white arrowheads), 12-French catheters (b-d, white arrows) were placed into the cysts. In cavitogram, there were lots of daughter vesicles (a and c, black arrowheads) within the cysts. In the first step, we destructed some daughter vesicles which located especially peripherally of the cyst that called “a liberated zone” (b, white circle). We continued the destruction of the daughter vesicles until there were no obvious daughter vesicles in the cavitograms (d)

After the first puncture by an 18 G Seldinger needle, a 0.035-inch Amplatz guidewire is advanced into the cavity and a 14F pigtail catheter is advanced over the guidewire. During the insertion of guidewire and catheter, some of the daughter vesicles are mechanically destroyed. Isotonic saline (0.9% NaCl) is injected into the cavity using 10 or 20 mL syringes and immediately aspirated at the same amount. During the process of injection and aspiration, some membrane fragments are also aspirated along with the fluid content by the catheter. This action is repeated multiple times (more than 100 if necessary) to evacuate as much content as possible. This type of irrigation was named as *“effective and aggressive irrigation of the cavity”* before and is named shortly as **“**effective irrigation”. It is important to create “a liberated zone” which defines the part of the cavity cleansed from the daughter cysts and degenerated membranes in the cavity around the tip of the catheter by irrigation (Fig. 1a-b). After cleansing a part of the cavity, the catheter needs to be moved to the neighbouring zone under fluoroscopic guidance. In most patients, it is not possible to have evacuation of all the content on the first session. Therefore, several sessions are employed until complete evacuation. After absence of any cysto-biliary fistula is confirmed by cavitography in the CE cysts whose daily drainage was less than 10 mL, hypertonic saline (30%) is introduced into the cavity for at least 6 min before complete evacuation. At the last step, ethanol (95%) is injected into the cavity and left in place for 5 min before evacuation and catheter removal. If it is not possible to reach eventual daughter cysts via a single catheter, catheter repositioning or extra catheter placement or PAIR for a daughter cyst within the mother cyst might be necessary before complete evacuation. The entire process of this technique was explained elaborately in previous studies [19, 20, 23].

When the daily drainage with bile continues over 200 mL for approximately 2 weeks, the patient is referred to an endoscopist to perform papillotomy in order to reduce the biliary pressure and achieve rapid closure of the fistula between the biliary tree and the mother cavity.

### Follow-up Protocol

Estimated cyst volume, fluid content and possible existence of daughter cysts were evaluated by US performed every 3 months during the first year, twice in the second year and annually thereafter if possible. Besides US examinations, CT and MRI studies were also acquired for further evaluation if needed. If recurrence or cysto-biliary fistula is suspected by ultrasonographic examination, MRCP is performed. If there is a suspicion of late abscess during follow-up, we prefer contrast-enhanced CT or contrast-enhanced MRI [24]. The estimated volumes were calculated by multiplication of three cyst diameters by 0.52. The positive treatment signs were considered reductions in cysts’ size and volumes, cyst wall thickening and irregularity, gradual decrease in fluid content and solidification [12]. Diagnosis of a daughter vesicle in the mother cyst or any other pathognomonic sign for viability was considered to be a recurrence.

### Statistical Analysis

As descriptive statistics for quantitative variables (age, cyst volume, duration of catheterization, length of hospital stay, etc.), if the distribution was symmetrical, the mean, minimum/maximum value and standard deviation were used; if the distribution was not symmetrical, the median, smallest/largest values were used. Number and percentage values were used for qualitative variables such as gender, cyst stage, major/minor complications and recurrence. SPSS for Windows (version 23.0, IBM SPSS Statistics, Chicago, IL) was used for the statistical evaluations.

## Results

### Main Characteristics

A total of 119 patients (F/M:73/59) with 132 liver CE2 and CE3b cysts were included into the study. Sixty-four (48.5%) CE2 cysts and 68 (51.5%) CE3b cysts were treated by Mo-CAT. Patients’ age ranges from 8 to 78 years (mean: 39 years). Twelve patients were under 18 years old. There was one CE cyst in 108 patients, two in 9 patients and three in 2 patients. As for cyst’s dimension, there were 13 cysts whose largest diameter was 5 cm or smaller than 5 cm, 40 cysts had at least one diameter larger than 10 cm, and the remaining cyst’s diameters were between 5 and 10 cm. While the dimensions of the smallest cyst were 39 × 26x20 mm, the dimensions of the largest one were 200 × 185x190 mm.

### Follow-up

Among all 119 patients, the mean follow-up duration was 51.66 ± 35.56 months (median, 49.00 months; range 0–131 months). Seven patients were lost of follow-up just after the procedure. Also, the follow-up was less than 6 months in 6 patients. When these 13 patients were excluded, the mean follow-up duration was 57.80 ± 32.75 months (median: 60.50 months; range 6–131 months) for 106 patients.

### Cavity Changes

The technical success rate was 100%. Before the Mo-CAT, the cyst volume was varied between 10.54 and 3655.60 mL and the mean cyst volume was 280.18 mL. The cyst volumes calculated at the last follow-ups varied between 0.00/686.40 mL with a mean of 72.41 ml. Among all patients, the mean reduction in the cyst volume during follow-up was 65.84% (range 6.29–100%). At the last follow-up, four cysts (3.0%) had totally disappeared.

### Complications

The complications encountered in the study were graded according to CIRSE classification system [22]. They were also divided into major/minor complications. Major complications were observed in 12 of 119 (10.08%) patients (Table 1).

**Table 1:** Major complications were encountered on 12 patients with no mortality

	Cysts (*n* = 132)	Patients (*n* = 119)	Papillotomy and biliary stent	CIRSE grade
Abscesses	2/132 (1.51%)	2/119 (1.68%)	-	3
Abscesses + CBF (Cysto-biliary Fistula)	5/132 (3.78%)	5/119 (4.20%)	5 (4.20%)	3
CBF and Recollection	1/132 (0.75%)	1/119 (0.84%)	1*(0.84%)	3
CBF	3/132 (2.27%)	3/119 (2.52%)	3 (2.52%)	3
Anaphylaxis	1/32 (0.75%)	1/119 (0.84%)	-	3
Perforation to the biliary system	-	-	-	-
*Total (%)*	*12/132 (9.09%)*	*12/119 (10.08%)*		

^*^Only ERCP (Endoscopic Retrograde Cholangiopancreatography)

### Major Complications

**Fig. 2:**
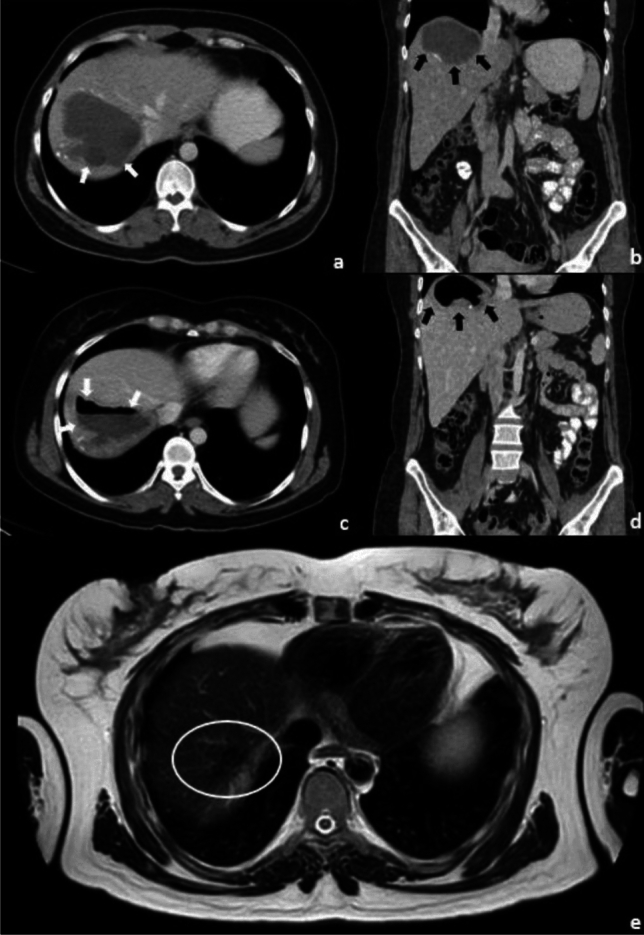
A 39-year-old female patient presented with partially peripheral calcified CE type 3b liver hydatid cyst (a and b, white/black arrows). After 9 days of the Mo-CAT procedure, in the abdominal computed tomography taken after the patient had a fever, there was an air-fluid level seen within the lesion (c and d, white/black arrows) consistent with abscesses. After effective drainage with 3 weeks and antibiotic therapy, there was no sign of active cyst or infection in T2-weighted MRI image obtained 1 year later (e, white circle) at the site of lesion but some scar tissue

Neither mortality nor abdominal dissemination was encountered in any patient. In 2 patients (1.68%), bacterial superinfection developed in the previously treated cysts cavity (Fig. 2). In 5 patients (4.20%), both CBF (cysto-biliary fistula) and abscess were diagnosed. The abscesses of 2 patients without CBF were successfully treated by percutaneous drainage and appropriate antibiotics. Five patients with CBF + abscesses underwent papillotomy + stenting of main bile duct on the day of 5, 14, 20, 40, 143 after Mo-CAT procedures. Therefore, the mean duration from Mo-CAT procedures to endoscopic procedures was 44.4 ± 56.59 days (median 20 days). Duration of the catheterization in these 5 patients was 65, 80, 90, 540, 0 days, respectively.

In 3 patients with only CBF (2.52%), daily drainage was more than 200 cc without infection. Endoscopic papillotomy + biliary plastic stents were performed on the day of 5, 17 and 21 after Mo-CAT besides keeping the drainage catheter within the cavity. Catheterization time which means duration of closing CBF was 135, 224 and 240 days (mean 199.36 days).

**Fig. 3:**
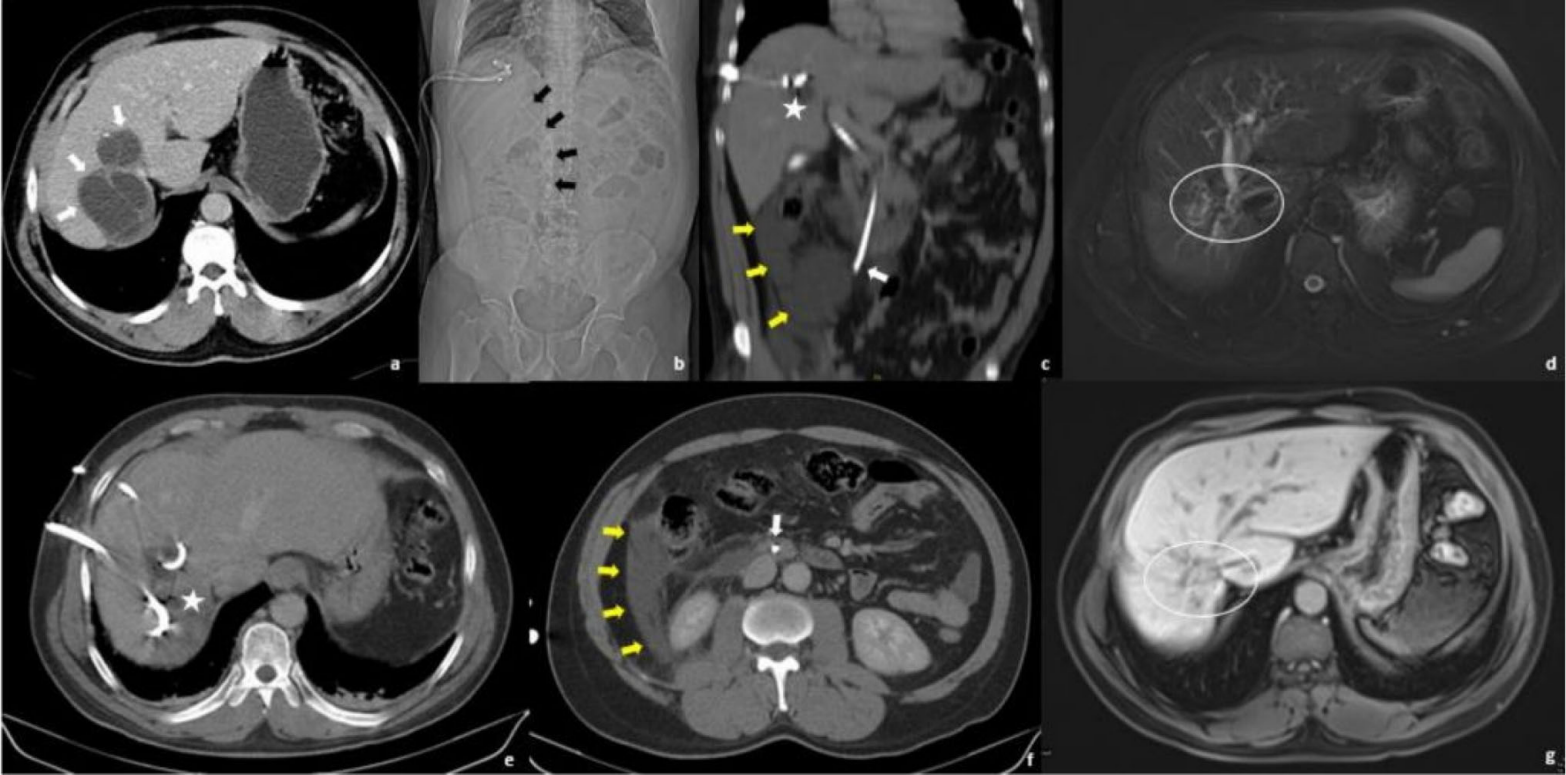
A 50-year-old male patient presented with bilobulated CE type 2 liver hydatid cyst (a, white arrows). During the procedure, cysto-biliary connection was detected at the cavitogram and the patient had bile-containing drainage from his catheter. ERCP was performed 5 days after the procedure, and a biliary stent was inserted. After 20 days, the patient presented with acute abdominal pain to the emergency room. It was obvious that some air bubbles (c and e, asterisk) within the cyst consistent with infection and lower tip of the choledochal catheter pass through the duodenal wall and extent to the right retroperitoneum (c and f, white arrows; b, black arrows); also there is some collection in there (c and f, yellow arrows). The ERCP catheter was removed, a catheter was inserted into the collection on the same day, and all these catheters were removed 5 weeks later when drainages were less than 5 ml per day. Two years later, control MRI revealed that there is no cyst or infection at these sites but some sequelae changes (d and g, white circles)

CBF and “Recollection” both were seen in 1 (0.84%) patient whose CBF was treated by only papillotomy at the beginning (Fig. 3). After diagnosis, recollection was treated successfully by percutaneous drainage + ethanol sclerosis. In 1 patient (0.84%), serious anaphylaxis developed during procedure was treated by appropriate medication by an anaesthesiologist. The patient was discharged uneventfully after 7 days.

### Minor Complications

Some features such as pain, fever, angioedema and pleural effusion occurred in 14 of 119 patients (11.76%) and resolved with only conservative treatment.

### Duration of Hospital Stay and Catheterization

The mean length of hospital stay was 3.88 ± 4.73 days (from 1 to 36 days/median: 2). A total of 107 (89.9%) of 119 patients discharged from the hospital in first week after Mo-CAT (2.71 ± 1.67 days). The mean length of hospital stay in 12 patients who stayed longer than a week was 14.33 ± 8.99 days. Mean duration of catheterization time was 15.84 ± 60.93 days which ranged from 0 to 540 days (median: 2). The catheters of 104 (87.4%) of 119 patients were removed in the first week after the procedure before discharge. In a small number of cysts (10 CE cysts), hypertonic saline and ethanol (95%) injections and evacuation were performed in the same session and the catheter was removed at the end of this session. Mo-CAT procedure was completed as a single session in these patients.

### Recurrence

Recurrence was detected in 6 (4.5%) of 132 cysts in 6 patients of 119 (5.0%) on US follow-up. Daughter cysts were demonstrated in the same cavity on the month of 11, 12, 17, 18, 20 and 81 months after Mo-CAT. Three of 6 cysts were successfully treated by a second Mo-CAT procedure. The fourth needed to undergo Mo-CAT procedure 2 more times before complete treatment. The fifth and sixth cysts had suspicious features for viability after the second Mo-CAT, but did not show any activity under Albendazole.

## Discussion

Modified catheterization technique (Mo-CAT) was first introduced in the early 2000s for the treatment of CE2/CE3b cysts and published later by our research team [19, 20]. Major goal of Mo-CAT is based on the evacuation of all cavity content including daughter cysts as well as the degenerated membranes. Evacuation is performed using only a 14F or smaller catheter by “*effective irrigation*” technique without using any other device [19–21]. Several studies have examined the outcomes of treating CE2/CE3b hydatid cysts using techniques like PAIR or S-CAT [11, 15, 17, 18, 25]. Although some cases were successfully treated with these techniques, recurrence rates were 46.6%, 47.8% and 61.5%, respectively [17–19]. Searching for a new technique for liver CE2/CE3b cysts had started at the beginning of 90s [20, 26–30]. A cutting device was invented to destroy all cyst content connected to a high-power suction apparatus to evacuate all content [28, 29]. Unfortunately, it was associated with serious major complications. Eight patients with nine Gharbi type IV liver cysts (CE 4) were successfully treated by using a 14-French Van Sonnenberg sump catheter by Haddad et al. [30]. In 2002, the percutaneous evacuation (PEVAC) was reported by Schipper et al. [26]. In this technique, content evacuation was provided with the help of a suction after a catheter insertion. However, major complications like CBF resulting in long catheterization time (mean: 53.6 days) and high cavity infection rate were reported [26]. Finally, searching for a novel technique to treat CE2/CE3b cysts ended up with the description of Mo-CAT with encouraging results [19–21, 31, 32].

The mean follow-up was 51.66 ± 35.56 months (median: 49.00/range 0–131) of all 119 patients, and the mean reduction in the cyst volume was 65.84% (range 6.29–100%). Volume reduction rate is similar to previously published study [19] as well as for CE1/3a cysts in the literature [11, 16, 25]**.** The duration of hospital stay was (mean: 3.88 days) also similar to previous studies [11, 20]. A total of 107 (89.9%) of 119 patients were discharged from the hospital in first week after Mo-CAT as prominent data besides the other 12 patients (10.1%) with major complications had longer duration than a week.

Major complications encountered in 12 patients (9.09%) were successfully treated by percutaneous techniques and/or endoscopic interventions. The most important major complications included CBF and cavity infections (11 patients) as they resulted in longer hospital stay and catheterization time.

The major complication rate in this study was dramatically lower than our earlier retrospective study (9.84% vs. 34.6%) because of the lower number of patients with CBF and cavity infections [19]. This results could be explained by two major reasons. Firstly, the sample size in the previous study was approximately 5-times lower than the sample size of this study. The second reason might be related to the increased experience of the physicians on Mo-CAT.

CBF/cavity infections’ rates in the current study are much lower than previously described other techniques [26, 29]. In other techniques, cavity content aspiration was generally obtained by using suction or similar devices. The techniques where suction is used are associated with high CBF rate resulting in longer catheterization time besides simultaneously high rate of cavity infection [33]. It can be argued that one reason for the high CBF rate is related to the evacuation technique, which results in a prolonged catheterization period and cavity infection. Therefore, “Effective irrigation” of Mo-CAT gives a chance to reduce CBF rate which means shorter catheterization time and lower cavity infection rate [19].

As a rule, catheters should remain in place until daily drainage decreases to less than 10 mL [19, 23]. If the daily drainage decreases gradually by giving an impression that it will cease in a short period of time, it could be wise to wait until the daily drainage stops. Otherwise, if the daily drainage does not stop in app. 10–14 days after the procedure, it seems wise to refer patients to the endoscopic papillotomy/stenting to reduce catheterization period as reduced catheterization period decreases the rate of cavity infection.

Surgical treatment of CE2/CE3b cysts is also associated with higher complication rates than surgery of CE1/CE3a cysts [33–35]. This was also reported the biliary complication rate after surgical treatment of hydatid cysts for Gharbi type III hydatid cysts (CE2/CE3b) 23.8%, while it was 12.5% for Gharbi I (CE1) [36]. In addition, surgical techniques are associated with higher mortality rates (2–4%) and morbidity rates (11–23%), as well as higher infection recurrence (2–10.4%) and longer hospitalization rates compared to PAIR [37]. In studies involving patients treated with different surgical techniques, the mortality rate is 0–1.2%, morbidity rate is 7.7–22%, and postoperative complication rate is 15–19%, which is higher than the percutaneous technique [38, 39]. In one of the most comprehensive reviews in the literature, which included 914 patients (1116 echinococcal cysts) who underwent surgical treatment for hydatid cysts, the authors concluded that the overall mortality rate was 0.22%, the morbidity rate was 15.07%, and the major complication rate was 15.07% [40].

Recollection of the cavity is mostly related to small CBF/CBFs which is generally invisible on fluoroscopy and easily treated by second drainage + ethanol sclerotherapy. Specifically, if there is no evidence for CBF on fluoroscopy, ethanol (95%) sclerotherapy is performed when daily drainage is less than 10 mL per day. Recollection associated with CBF occurred in one patient (0.7%) treated by drainage + sclerotherapy.

We carried out all percutaneous CE treatment under general anaesthesia to avoid patients’ sudden death because of serious anaphylaxis/anaphylactic shock. In one patient (0.7%), anaphylactic shock was developed and treated by appropriate medication by anaesthesiology team.

Recurrence rate (4.5%) encountered in 6 cysts in 6 patients was almost similar to previously published rate (3.8%) [19]. It is generally accepted that the recurrent cysts are treated percutaneously by second Mo-CAT.

The most important limitation of this study is the single-arm/single-centre study rather than a comparative study. In addition, there are 7 patients who were lost during follow-up.

Strength of this study is related to be a large cohort study in the literature to evaluate safety, efficacy, and long-term results of the patients with liver CE2/CE3b cysts treated by only Mo-CAT.

In conclusion, Mo-CAT is a safe and effective treatment technique for liver CE2/CE3b cysts associated with acceptable complication and recurrence rates. Therefore, it can be accepted to be a valid alternative to surgery. Although we suggest that CE2/CE3b cysts should be treated with Mo-CAT as a first choice instead of surgery, a prospective randomized trial is required to compare the results of Mo-CAT and surgery. However, more evidence is needed in the literature for this suggestion to be acceptable.
